# Supplementary material for: Traces of Parenthood but Not Pregnancy Loss in UK Biobank Structural Brain MRI Data
Source: Hum Brain Mapp. 2026 Apr 12;47(5):e70527. doi: 10.1002/hbm.70527 (PMC13070878; doi:10.1002/hbm.70527)
Supplement: Supplementary file 1 — Data S1: Supporting Information. Figure S1: Comparison of t(parenthood) values from the main analysis and supplemental analysis, controlling for SES. Figure S2: Distributions of t(motherhood), t(fatherhood) and t(loss) from supplementary analysis, omitting age matching. T values result from linear regression models in the Female Full and Male Full dataset. Table S1: Raw (unchanged sign) t(parenthood) values for all regions significantly affected by parenthood in either sex. Table S2: XGBoost parenthood classifier hyperparameters tested in grid search, and final hyperparameters for both models, selected based on highest ROC‐AUC. [file HBM-47-e70527-s001.docx]

# Supplemental material

**Univariate analysis**

We performed an additional analysis including the “Townsend deprivation index” as a measure of socioeconomic status (SES) as a covariate. In the Training and Testing Set of Females as well as the Training Set of Males (see Table 1), also used in the main analysis, we thus computed

$$V\left( ROI \right)\sim parenthood+townsend\_index+loss+age+eTIV+headmotion+site$$

For easier comparisons, we here show the effects of parenthood from the main analysis, compared to the effects of parenthood when controlling for SES in regions significantly affected in either main analysis, similar to Figure 1B.


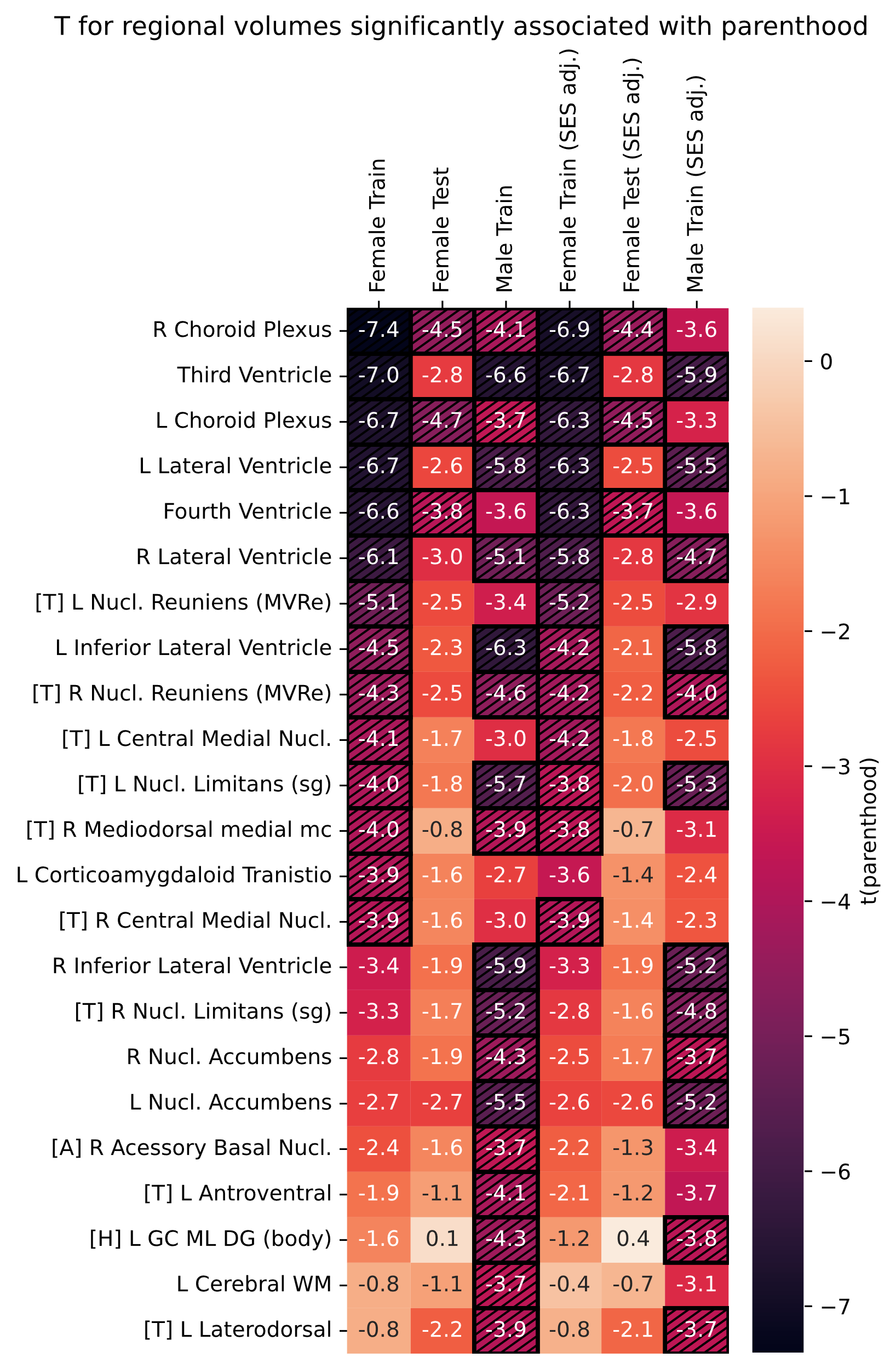


Supplemental Figure 1: Comparison of t(parenthood) values from the main analysis and supplemental analysis, controlling for SES.

As expected, the effect statistic is slightly smaller for the majority of regions when controlling for SES. However, overall we find a very similar pattern of effects compared to our main analysis.

We performed an additional analysis to make use of our full dataset without our rigorous age-matching procedure. In our Full Dataset of men (see Table 1), we computed the effects of *fatherhood* using the same linear regression model as described above (see Methods). Similarly, we computed the effects of *motherhood* and pregnancy *loss,* however, in the same linear model, thus calculating

$$V\left( ROI \right)\sim parenthood+loss+age+eTIV+headmotion+site$$

The resulting distributions of *t(motherhood), t(fatherhood)* and *t(loss)* are shown in Supplemental Figure 2.


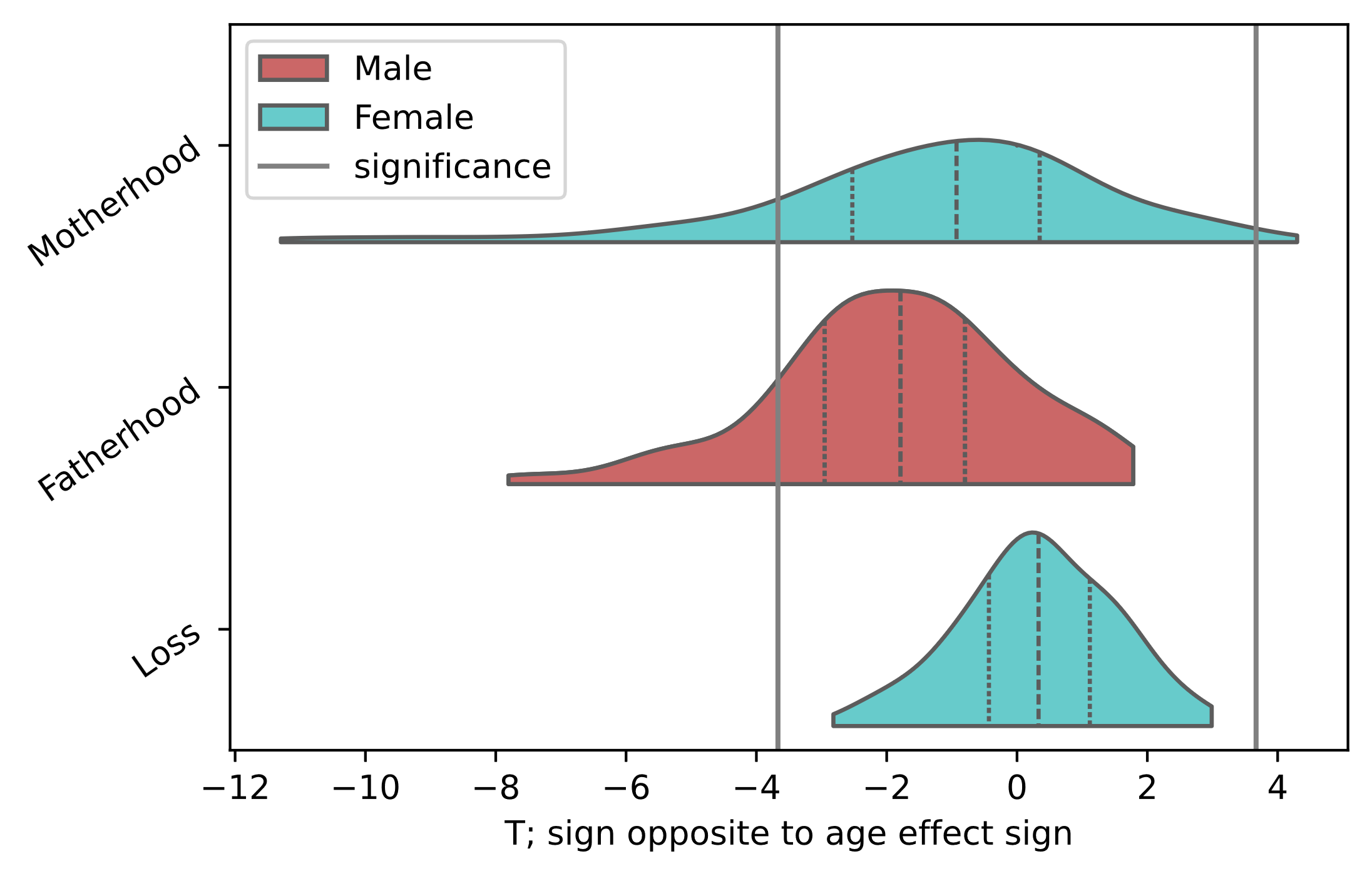


Supplementary Figure 2: Distributions of t(motherhood), t(fatherhood) and t(loss) from supplementary analysis, omitting age matching. T values result from linear regression models in the Female Full and Male Full dataset.

Similarly to our main analysis, we find negatively shifted distributions of *t* values for motherhood and fatherhood and numerous brain regions surpassing significance level. This pattern is more pronounced compared to our main analysis. Different from our main analysis, there are brain regions where the motherhood effect surpasses significance where the motherhood effect acts in the same direction of age. The distribution of *t(loss)* is shifted towards positive values, underscoring no neuroprotective effects of lost pregnancies could be identified. In this analysis, *t(motherhood)* and *t(fatherhood)* correlate with Pearson’s r = 0.6.

Figure 1B in the main manuscript shows effect statistics for all brain regions significantly associated with parenthood in either sex, respective to the age effect. Here, Supplemental Table 1 shows raw effect statistics for the same regions.

| **Region** | **t(motherhood), Female Train** | **t(motherhood), Female Test** | **t(fatherhood), Male Train** |
| --- | --- | --- | --- |
| R Choroid Plexus | -7.4 | -4.5 | -4.1 |
| Third Ventricle | -7.0 | -2.8 | -6.6 |
| L Choroid Plexus | -6.7 | -4.7 | -3.7 |
| L Lateral Ventricle | -6.7 | -2.6 | -5.8 |
| Fourth Ventricle | -6.6 | -3.8 | -3.6 |
| R Lateral Ventricle | -6.1 | -3 | -5.1 |
| [T] L Nucl. Reuniens (MVRe) | 5.1 | 2.5 | 3.4 |
| L Inferior Lateral Ventricle | -4.5 | -2.3 | -6.3 |
| [T] R Nucl. Reuniens (MVRe) | 4.3 | 2.5 | 4.6 |
| [T] L Central Medial Nucl. | 4.1 | 1.7 | 3.0 |
| [T] L Nucl. Limitans (sg) | -4.0 | -1.8 | -5.7 |
| [T] R Mediodorsal medial mc | 4.0 | 0.8 | 3.9 |
| L Corticoamygdaloid Trans. | 3.9 | 1.6 | 2.7 |
| [T] R Central Medial Nucl. | 3.9 | 1.6 | 3.0 |
| R Inferior Lateral Ventricle | -3.4 | -1.9 | -5.9 |
| [T] R Nucl. Limitans (sg) | -3.3 | -1.7 | -5.2 |
| R Nucl. Accumbens | 2.8 | 1.9 | 4.3 |
| L Nucl. Accumbens | 2.7 | 2.7 | 5.5 |
| [A] R Accessory Basal Nucl. | 2.4 | 1.6 | 3.7 |
| [T] L Anteroventral | 1.9 | 1.1 | 4.1 |
| [H] L GC ML DG (body) | 1.6 | -0.1 | -4.3 |
| L Cerebral WM | 0.8 | 1.1 | 3.7 |
| [T] L Laterodorsal | 0.8 | 2.2 | 3.9 |

Supplemental Table 1: Raw (unchanged sign) t(parenthood) values for all regions significantly affected by parenthood in either sex.

**Machine learning analysis**

In our machine learning analysis, we performed a parameter grid search, building models with different variations of hyperparameters for the XGBoost classifier model. We then measured performance by 10-fold cross validation on the training set. The tested hyperparameter grid and the final values resulting in the best performance are shown in Supplemental Table 1.

| **Parameter** | **Values in Grid Search** | **Value in final Motherhood Model** | **Value in final Fatherhood Model** |
| --- | --- | --- | --- |
| eta | 0.001, 0.005, 0.01, 0.05, 0.1 | 0.001 | 0.05 |
| n_estimators | 25, 50, 100, 200, 400 | 400 | 50 |
| subsample | 0.6, 0.8, 1.0 | 0.6 | 0.6 |
| colsample_bytree | 0.6, 0.8, 1.0 | 0.6 | 0.6 |
| max_depth | 2, 3, 5 | 5 | 3 |
| min_child_weight | 0.5, 1, 3 | 3 | 3 |

*Supplementary Table 2: XGBoost parenthood classifier hyperparameters tested in grid search, and final hyperparameters for both models, selected based on highest ROC-AUC.*
